# Approaching the Target: the Path Towards an Effective Malaria Vaccine

**DOI:** 10.4084/MJHID.2012.015

**Published:** 2012-03-10

**Authors:** Alberto L. García-Basteiro, Quique Bassat, Pedro L. Alonso

**Affiliations:** 1 Preventative Medicine and Epidemiology Unit, Hospital Clínic, Barcelona, Spain; 2 Barcelona Centre for International Health Research (CRESIB, Hospital Clínic-Universitat de Barcelona), Barcelona, Spain

## Abstract

Developing an effective malaria vaccine has been the goal of the scientific community for many years. A malaria vaccine, added to existing tools and strategies, would further prevent infection and decrease the unacceptable malaria morbidity and mortality burden. Great progress has been made over the last decade and a number of vaccine candidates are in the clinical phases of development. The RTS,S malaria vaccine candidate, based on a recombinant *P. falciparum* protein, is the most advanced of such candidates, currently undergoing a large phase III trial. RTS,S has consistently shown around 50% efficacy protecting against the first clinical episode of malaria, in some cases extending up to 4 years. It is hoped that RTS,S will eventually become the first licensed malaria vaccine. This first vaccine against a human parasite is a groundbreaking achievement, but improved malaria vaccines conferring higher protection will be needed if the aspiration of malaria eradication is to be achieved.

## Introduction

Malaria is considered the most important parasitic disease in the world. It is one of the ten leading causes of death in low income countries. During 2010, it was estimated that malaria caused around 216 million clinical episodes worldwide, being responsible for 655,000 deaths, the majority of which in African children.^1^ In addition, it also contributes to impoverish local economies and consumes substantial health resources. Providing a comprehensive set of malaria control interventions to reduce incidence and mortality is projected to cost around 3 US billion dollars per year in Africa alone.^2^

There has been a significant increase in funding malaria control interventions and research over the last decade. In consequence, many countries have adopted evidence-based interventions to fight malaria, such as indoor residual spraying (IRS), long lasting insecticidal nets (LLIN), rapid diagnostic tests (RDT) and arteminisin-based combination therapies (ACTs). Intermittent Preventive Treatment in Pregnant Woman and Infants (IPTp and IPTi respectively), which have demonstrated to be effective in clinical trials,^3^–^4^ are innovative strategies using drugs for prevention which are currently recommended by WHO in areas of high transmission, albeit their implementation being variable and in some cases (IPTi) inexistent.

Despite all the efforts made in the use and implementation of such malaria control interventions, it is thought that in order to decrease substantially the burden of disease and advance towards the aspiration of malaria eradication, effective vaccines against malaria are needed and should play a crucial role.^5^ Despite the morbidity and mortality burden attributable to malaria, there are other factors that make an effective malaria vaccine desirable. The resistance profile of malaria parasites to an increasing number of antimalarial drugs and readily available insecticides, the unequal and inadequate distribution of malaria control tools in different settings or the increased movement of migrant populations and tourists to endemic areas are important arguments in favour of concentrating resources towards malaria vaccine research. However, despite current advances towards getting a effective malaria vaccine, scaling up available malaria control interventions seems the current realistic strategy to reduce the health burden of malaria,^6^ and in many cases may probably be sufficient to approach the desired elimination goal.^7^ The most advanced malaria vaccine, the RTS,S candidate, is currently undergoing a pre-licensure trial, and although it constitutes a historical advance in malaria research, more effective second generation vaccines will be required.

## Brief History of Malaria Vaccines

The quest for an effective malaria vaccine has been a goal for the scientific community over many years. Studies with different species of malaria in rodents and birds have been conducted since 1910.^8^ However, the most significant advances have occurred over the last 50 years. Studies by Nussenzweig *et al* in the 60’s showed protective immunity in rodents after injecting irradiated rodent sporozoites.^9^ Subsequent studies identified the circumsporozoite protein (CSP), a surface protein of the sporozoite, as an important target for antibodies.^10^ In the seventies, Clyde and colleagues showed in humans that it was possible to obtain protection against infection after multiples bites of irradiated sporozoites.^11^ From the 1980s onwards, major focus was given to identify different sporozoite surface antigens, potential targets of monoclonal and polyclonal antibodies. Some of them were part of early vaccine candidates, although not demonstrating significant protection.

In the 1980s, researchers from South America developed an asexual blood stage peptide-based vaccine (SPf66), which seemed to be efficacious in monkeys and humans.^12^ However, subsequent studies conducted in different malaria endemic countries, failed to provide similar results.^13^–^14^ At about the same time, GlaxoSmithKline (GSK) conducted different studies using different peptides after sequencing the epitopes of protective antibodies directed to the central region of *P. falciparum’s* CSP.^15^ From all the different formulations, the construct that obtained better results in preclinical trials was one made with the hepatitis B virus surface antigen, called RTS,S.^16^ This construct was tested with different innovative adjuvants, AS04, AS03, AS02 and AS01, the latter showing the best results^17^ and currently undergoing a large phase III trial in different settings in Africa.

In parallel to the development of the first RTS,S vaccines, different studies evaluated the response to vaccines containing different peptides and proteins of the asexual blood stage of the parasite, such as the apical membrane antigen 1 (AMA1), merozoite surface protein (MSP) 1, MSP2, MSP3, among others.^18^ None of these vaccines have, however, shown as of today a satisfying clinical protection similar to the positive results shown by RTS,S. At the end of the 90’s new vaccines aiming to enhance cellular immunity response, such as DNA vaccines and peptide vaccines, were developed, although they have not yet proven to be efficacious in humans^19^. In recent years, new viral vectored vaccines have been developed in animal models obtaining promising results,^20^–^21^ and now need to be assessed in malaria endemic countries, where many vaccines have failed to demonstrate efficacy.

## Desirable Characteristics for a Malaria Vaccine

The main desirable characteristics for a malaria vaccine should include a good safety profile together with a high efficacy against malaria infection and disease. Nonetheless, there are other many desirable conditions that need to be addressed together with malaria vaccine development. Most of them are depicted in table 1. Apart from the abovementioned characteristics, the scientific community and decision makers have to ensure that effective and safe vaccines reach the target populations they have been intended to. As it has been suggested ensuring that “the limits of science are not constrained by the limits of systems" (excerpt by Dr Orin Levine, Johns Hopkins Bloomberg School of Public Health)^22^ must be an imperative when thinking of vaccines whose main beneficiaries live in low income countries with weak deployment mechanisms.

## How Malaria Vaccines Work

To understand the different approaches and targets used in preclinical and clinical malaria vaccine research, it is crucial to understand the different stages of the parasite within the infection cycle.

### The infection cycle

**Figure 1:**
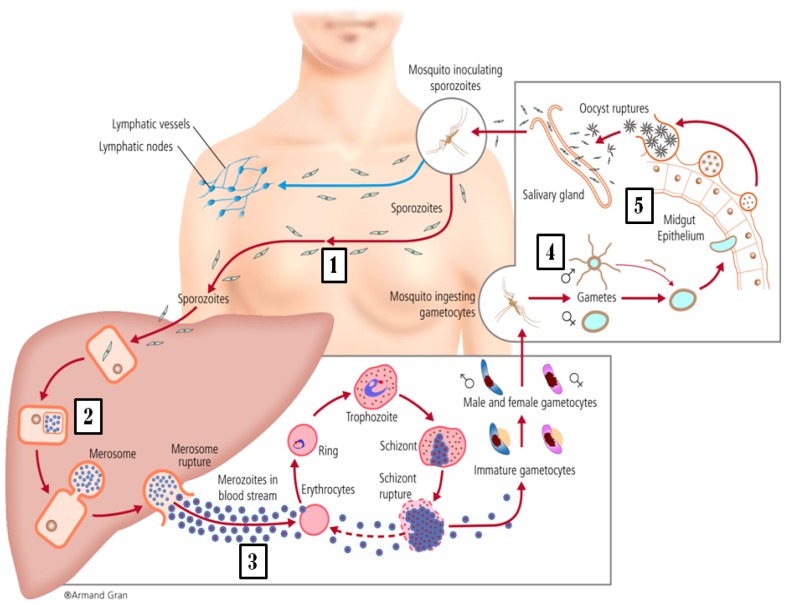
*Plasmodium falciparum Life Cycle.* The different targets of malaria vaccines. (1) Sporozoites targetted by pre-erythocityc vaccines), (2) Liver stages of parasite targetted by pre-erythrocytic vaccines, (3) Asexual blood stages, mainly merozoites. (4) Parasite sexual stage in the mosquito midgut and (5) midgut wall antigens (vector-stage vaccines, indirectly acting against ookinete stage of the parasite).

The parasite gets into human blood through the bite of a female mosquito *Anopheles*. Once in the blood of the infected person where it will only circulate for a few minutes, the parasite rapidly migrates to the liver, infecting hepatic cells, where for a few days (incubation period) it multiplies and grows (liver stage). Then it is released into the blood stream, where they infect red blood cells (blood stage). In the blood stage they multiply and differentiate causing clinical symptomatology, before being picked up again in a mosquito bite. The parasites can be found at different stages inside the human body. The main ones are: sporozoites, when they are free in the blood after being injected by the mosquito, schizonts and merozoites within the liver and in the blood, and gametocytes in the blood, which are sexually differentiated stages picked up by the mosquito during the blood meal and thus the only stages of the parasite responsible for transmission to the mosquito and the next human being. Inside the mosquito, the parasite also adopts different stages during a cycle of growth: gametes, right after they have been picked up in the blood meal and deposited in the mosquito midgut lumen where they are fertilized; ookinetes, which are the evolution of the zygote produced by the fusion of gametocytes; oocysts, the next stage that continues developing in the epithelium of the midgut; and finally sporozoites, which are the product of a mature oocyst. They migrate to the salivary glands from the mosquito midgut, and these parasites are ready to infect the next human, perpetuating the life cycle (figure 1).^23^

### Why is it so difficult to obtain a malaria vaccine?

The absence of immune correlates of protection for malaria vaccines has been a longstanding obstacle in malaria vaccine development. The discovery of a biomarker which could behave as a reliable proxy of protection against clinical disease, together with the availability of a predictive animal model (currently existing, but suboptimal) would make development efforts much easier and more efficient. Many knowledge gaps will certainly need to be filled regarding naturally acquired immunity and its molecular and epidemiological determinants.

The complexity of the parasite (as compared to viruses or even bacteria) is clearly another limiting factor. Any of the malaria plasmodia present thousands of antigens, which differ depending on the parasite stage of the cycle in both the human host and vector. Moreover, the immune responses against different stages of the parasite have been proven to vary, hindering the possibility of finding those who play a major role triggering human immunity, which would be the desirable candidates for a malaria vaccine. In addition, many antigens expressed by the parasite are highly polymorphic within the same host, adding, if possible, more complexity to the already difficult process of antigen identification. In conclusion, no vaccine against parasites has been licensed yet, and this is not circumstantial.

## Malaria Vaccine Approaches

Different classifications for malaria vaccine candidates have been use by different authors. They are based on the phase of the parasite cycle they mainly target (and hence the expected disease outcome), the composition of the vaccine itself (whole organism, recombinant proteins or DNA, and some of them adjuvated or vectored), the vaccination strategy (different dose schedules, prime and boost regimes), the capacity to interrupt transmission (irrespective or not of their effect on clinical disease) and the population they are intended to reach (population in endemic areas, travellers). However, some of the vaccine candidates act in different stages of the parasite combining different strategies, making difficult to create non-overlapping categories for any particular classification. The most frequently used classification, which splits vaccine candidates into pre-erythrocytic, blood stage and transmission blocking vaccines,^24^–^26^ seems somewhat out of date for several reasons. First, effective pre-erythrocytic and asexual (blood) stage vaccines would have an impact on transmission,^5^ because they would dramatically decrease parasitaemia, having consequently room within the “transmission blocking category”. Second, the term “blood stage” is somewhat broad, and does not really specify whether they target the sexual or asexual stage of the parasite. Therefore, “asexual blood stage” is probably a more accurate term. Third, candidates whose main targets are sexual stage parasites or even mosquito wall cells, deserve a category on their own.

Moreover, the impact of a vaccine targeting a specific part of the cycle may transcend its stage specificity. For instance, a pre-erythrocytic vaccine, such as RTS,S, conferring partial pre-erythrocytic immunity may also induce blood (asexual) stage immunity by allowing the passage of some parasites (“leaky” vaccine) to the blood. Such low dose parasitaemia reaching the blood would contribute to the generation of a local (i.e. blood stage) immune response, protective against clinical disease.^27^ However, since the main effect of this vaccine is the induction of antibodies against pre-erythrocityc specific antigens, pre-erythrocytic vaccines as a category is probably still a valid and clearly differentiated category on its own. In this review, vaccine candidates are classified depending on the life cycle stage they mainly act against.

## Pre-erythrocytic Vaccines

These vaccines are indented to elicit an immune response against antigens of the initial stages of the infection, i.e. antigens exposed from the entrance of parasites into the blood (sporozoites) or hepatic stages before the parasite exits the liver to the blood. If a vaccine designed to follow this strategy were 100% effective, it would completely block the infecting parasites from reaching the blood stages, avoiding therefore any clinical symptomatology and risk of subsequent transmission. Thus, these malaria vaccines could conceptually protect from any malaria episode not only children and pregnant women living in malaria endemic countries, but also those who have not been exposed to malaria, such as travellers, since they are at higher risk of severe infection. However, if efficacy was partial and not complete, as phase II and III trials seem to show, the effect of vaccination would include a decrease in the parasite load which is released into the blood from the liver, thus minimizing (but not averting) the effect of a malaria infection.

The first attempts to obtain a malaria vaccine were precisely based in this concept, targeting through attenuated sporozoite challenges in different animals the pre-erythrocytic stages of malaria infection^9^. In further studies, naïve volunteers were then exposed to UV irradiation-weakened sporozoites, obtaining a complete immunity in 90% of the cases, although for a short period of time.^28^ This early studies aiming to obtain pre-erythrocityc immunity, made scientists believe that a malaria vaccine could be possible. Similarly, the RTS,S malaria candidate vaccine, also pre-erythrocytic by design has, for the time being, shown the most promising results, and is leading the race for being the first malaria vaccine licensed in the near future.

**Figure 2:**
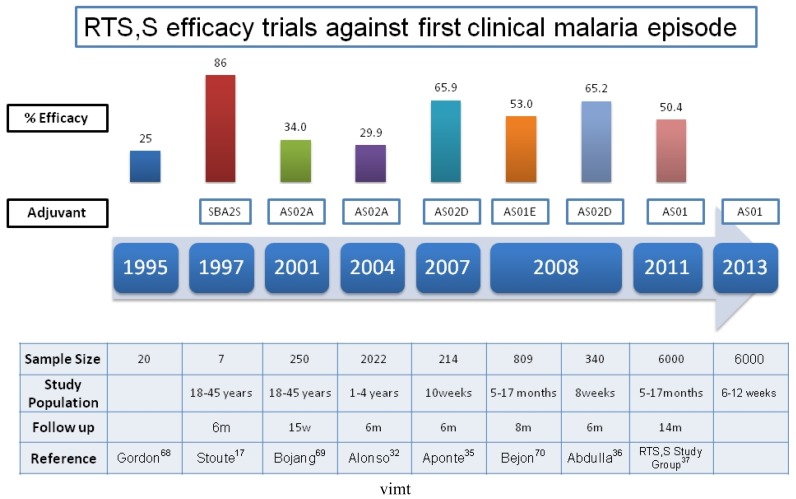
*RTS,S efficacy trials against first clinical episode.* Note that efficacy point estimates have been assessed at different follow up intervals. This chart includes only the first efficacy results of each project.

The RTS,S malaria vaccine uses a construct made of the central repeat region of the *P. falciparum* circumsporozoite protein (CSP) and the hepatitis B surface antigen (HBsAg) as a carrier. It is therefore a recombinant subunit vaccine. Much of its success is attributed to the adjuvant systems used in this formulation: AS02 and AS01. The first phase I trial in young adults in endemic areas was conducted in The Gambia, showing a good safety and immunogenicity profile with a three dose schedule,^29^ but with a limited duration of protection. Same phase trials in children using RTS,S/AS02 were conducted in The Gambia (children 1–11 years old), with same positive results as in adults.^30^–^31^ Therefore, a phase IIb efficacy study was conducted in 2022 Mozambican children aged 1 to 4 years, showing efficacy against clinical malaria (first episode) of around 30%; against malarial infection of 37%, and against severe malaria of around 58%.^32^–^33^ Follow up of participant children confirmed that efficacy was maintained at least up to 45 months.^34^ As a logical follow-up considering that the ideal target population should be young infants, another trial showed that the RTS,S/AS02D malaria vaccine was safe, well tolerated and immunogenic when administered to young infants in Mozambique on a vaccination schedule staggered with other vaccines belonging to the EPI schedule.^35^ Trials administering the vaccine to infants simultaneously to the rest of EPI vaccines showed no apparent interferences and good and similar safety and efficacy profiles.^36^ Led by these promising results, a large phase III trial with RTS,S/AS01 was initiated in 11 sites in 7 African countries including 15,460 children of two different age categories: 6 to 12 weeks and 5 to 17 months of age. The preliminary results of this trial have confirmed the encouraging results obtained over the last decade in earlier stage trials, with efficacy estimates against first malaria episode of 50.4%, and against severe malaria of 45.1% (in the older cohort). The combined efficacy against severe malaria in both age categories (children 6 weeks to 17 months) was 34.9%. There were no differences in the safety profile of malaria vaccinated children compared with the control group.^37^ Further results on safety and immunogenicity of this vaccine will be published by the end of the study which has been scheduled in 2013. (Figure 2) This study, cofunded as the earlier trials of RTS,S by GSK, and the Malaria Vaccine Initiative (MVI) at PATH, has been the largest trial ever conducted in children in this continent.

Other attempts to obtain a vaccine inducing pre-erythrocytic with whole parasites (sporozoites) in the past and a renewed interest has recently been observed in these types of vaccines. An early study consisted of exposing healthy volunteers to the inoculation of radiation-attenuated *P. falciparum* sporozoites by mosquito bites.^38^ It showed a 93% protection, although the number of subjects in this trial was limited (only 14 subjects). Another recent study made in the Netherlands tried to induce immunity in challenged subjects through the inoculation of intact sporozoites by means of mosquito bites while receiving a prophylactic regimen of chloroquine.

An homologous challenge after discontinuation of chloroquine showed the induction of cell mediated protective immunity.^39^ A further study suggests that this protection might last up to 2 years after artificial immunization.^40^

The approach of an American biotech company using the whole parasite strategy was to develop a vaccine that could be administered applying the same principles of radiation-attenuated *P. falciparum* sporozoites (since exposing vaccine recipients to mosquito bites is not a realistic option).^41^ They obtained a vaccine using parasites from mosquito’s salivary glands, irradiated them and subsequently criopreserved them in liquid nitrogen. The first phase I clinical trial began in May 2009 and included the skin inoculation of this vaccine (known as PfSPZ) to 80 healthy US volunteers, but failed to show the expected protection seen in historical trials.^42^ Newer possibilities to mimic the effect of irradiated sporozoites through mosquito bites could be the development of an intravenous vaccine or the recent strategy using non irradiated genetically attenuated parasites, but these alternatives still need to be explored.

### Prime boost vector malaria vaccines

This is an innovative and promising strategy which mainly targets liver stage antigens through the combination of different regimes and vaccine composition. Several studies in animal models have been conducted using different mixtures of prime and boost immunization combinations using viral vectors which express different *P. falciparum* antigens and DNA plasmids (encoding the same antigen expressed in the viral vector). A few antigens (mainly pre-erythrocytic) have been targeted by both viral vectors and DNA plasmids used alone or in prime boosting regimes, although the thrombospondin-related adhesive protein (TRAP) has conferred the best immunogenicity. Likewise, several viral vectors (vaccinia virus, fowlpox, modified Ankara virus, adenovirus) have been tried out.^43^ The use of single DNA plasmids encoding TRAP is limited since they have only demonstrated to induce CD8 immunity in animal models failing to do so in humans^44^. However, viral vectors have been shown to induce both humoral and cell mediated immunity.^21^, ^45^ A further extension by Draper and colleagues in animal models have shown that a prime boost strategy (including adenovirus type 5 and Modified Vaccinia Virus Ankara(MVA) expressing MSP-1) regime is capable of inducing powerful blood stage antibody responses and can elicit a cell mediated response while reducing the parasite load at the liver.^20^,^46^ At the moment, the leading vaccine using a prime boost regime (which includes a heterologous Chimpanzee Adenovirus and a MVA) is entering a phase Ib safety and immunogenicity trial in Africa.^43^ Much is expected from these vaccine platforms, although the critical trials in malaria endemic countries still need to confirm what has been found in the early studies.

## Asexual Blood-Stage Vaccines

Asexual blood stage vaccines are those whose objective is preventing the erythrocyte invasion and blocking the infected red blood cells from adherence to several tissues, which is indeed the onset of a clinical malaria disease. They would not be effective towards preventing infection; they would rather mitigate the clinical symptoms of a malaria episode. Some authors suggest that this type of vaccines would need to be combined in a multicomponent vaccine with perhaps pre-erythrocityc or sexual stage components. It is known that natural immunity against blood stage antigens can be induced,^47^ and perhaps it might also play a major role in the long lasting protection shown by RTS,S malaria vaccine, due to the occurrence of “leaky” immunity when pre-erythrocytic immunity wanes.^27^

Most asexual stage vaccine candidates target the induction of antibody response against merozoite antigens. The targeted antigens which have been evaluated for impact in malaria exposed children are the apical membrane protein (AMA1), merozoite surface protein 1 (MSP1), 2 (MSP2) and 3 (MSP3), the glutamate-rich protein long synthetic peptide vaccine (GRURP), the ring-infected erythrocyte surface antigen (RESA), serine repeat antigen (SERA5) and erythrocyte-binding antigen 175 (EBA 175).^48^ Most clinical trials have not shown significant impact on preventing clinical malaria although some of them have shown to reduce parasite density.^49^–^50^ The absence of correlates of protection and antigenic variation of blood stage antigens still are some of the major problems of this type of vaccines.^18^

The results of a field trial conducted in Mali with a malaria vaccine based on AMA1 have been recently published. Efficacy against the primary endpoint (clinical malaria) was poor, only 17.4% (hazard ratio 0.83, 95% CI 0.63 to 1.09), although the strain specific-efficacy (against malaria caused with parasites with AMA1 included in the vaccine) was much higher, suggesting that it might be worth considering this construct for a multicomponent malaria vaccine.^50^

Another innovative strategy to induce asexual blood stage immunity implies the inoculation of red blood cells with whole attenuated merozoites, in order to provoke a cell mediated response. This challenge trial has been successfully conducted by Pombo *et al* in Australia.^47^ They challenged 5 healthy subjects by inoculating infected red blood cells followed by drug cure. In the 4^th^ round, before the drug cure, they could not find any parasites or antibodies in the blood of these subjects, but rather a proliferative T cell response involving CD4 and CD8 cells.^47^,^51^ Further trials with larger amount of subjects are needed to confirm these results if some logistic and technical issues involving the use of human blood and growth of merozoites can be overcome.

## Sexual Stage Vaccines

This category would include most of the classical transmission blocking vaccines, term now embraced by the recent concept of “vaccines that interrupt malaria transmission” (VIMT). Vaccines that target the sexual stage of *Plasmodia* would not protect the vaccinated recipient from a malaria episode, but rather the community the subject belongs to (reason why they were also called “altruistic” vaccines). These vaccines would avoid the fertilization process of the gametes or the invasion in the mosquito midgut, and prevent from further development in the vector, therefore interrupting the transmission of the disease to the subsequent individuals. Although the immune mechanisms have not yet been fully understood, it is thought that the sexual stage specific antibodies, together with cytokines and the complement, would be taken by the mosquito in the blood meal, interfering with the formation of a new oocyst in the vector midgut.^52^ There are four main antigens that have been targeted in vaccine preclinical trials: P230, P48/45, which are proteins from the gametocyte and P28 and P25 which are proteins from the zygote or ookynete stage.^52^ The latter, P25, is the target of the only sexual stage candidate that has reached the clinical phase of development. However, should these candidates be considered for the interruption of transmission, they would need to be applied to a large proportion of the population of the area (since any infected person would be capable of transmitting malaria), raising further technical and regulatory issues.

### Vaccines against vector molecules

Another vaccine project that would have a high impact on transmission of any Plasmodium species would be a vector stage/ookinete stage vaccine targeting mosquito’s midgut molecules, which are essential for parasite growth within the mosquito.^53^ It has been recently shown that ookinete invasion could be inhibited by antibodies against aminopeptidase N (AgAPN1), a midgut surface antigen^54^. Nonetheless, in case an effective vaccine based on mosquito midgut antigens could be developed, major deployment complexities would also need to be addressed.

## Other Vaccines

### Vaccines against P Vivax

Although greater efforts have been dedicated to vaccines against *P. falciparum*, there is a growing interest on eliciting a vaccine against *P. vivax*, the most widespread malaria species in the world, especially in South and Central America, certain Middle Eastern countries and Asia and the South Pacific. *P. vivax* is not as deadly as *P. falciparum*, but causes considerable morbidity and consumes not few health resources. Several difficulties have been encountered to develop *P. vivax* vaccines. Besides the lack of resources allocated for *P. vivax* vaccine research, there have been few *P. vivax* target antigens identified with vaccine potential, as compared to *P. falciparum*: the CS protein, SSP2/MTRAP, the Duffy binding protein, the MSP1, the AMA1, P25 and P28.^55^ The different behavior of the parasite within the human host and the probably different immune response induced by *P. vivax* (compared to *P. falciparum*) adds a lot of complexity to vaccine development.^56^ There have been several preclinical trials and sporozoite challenge trials with *P. vivax* vaccines but up to our knowledge, there have been few phase I clinical trials (no phase II trials yet). The two main targets tested at phase I clinical phase are *P. vivax* circumsporozoite (CS)^57^–^58^ and P25.^59^–^60^ There is an ongoing phase I/IIa trial based on a CS protein with the AS01B adjuvant currently being conducted in malaria naïve individuals by the U.S. Army Medical Research and Materiel Command (USAMRMC)

### Malaria vaccines for pregnancy

Most malaria vaccine candidate trials entering clinical phases in malaria endemic countries have taken children as study population. The rational is that they are the most vulnerable group for malaria, enduring higher morbidity and mortality. In case these vaccines provide lifelong protection, greater advantage would be obtained by vaccinating at early stages of life. In case the RTS,S malaria vaccine is licensed, it would probably be delivered with other childhood vaccines. However, in case the duration of protection is shorter, it is reasonable to think that periodical or boosting vaccination would be needed to protect populations at different stages of life. The other major high risk group for malaria, apart from children, towards which greater efforts need to be made to guarantee protection, are pregnant women, who are highly vulnerable to the disease in endemic areas. The existing knowledge gaps in malaria in pregnancy immunology and pathogenesis adds complexity to the objective of eliciting effective vaccines to provide protection to mothers, fetus and newborns.

One of these gaps involves the binding of parasitized erythrocytes to the chondroitin sulfate A (CSA), a glycosaminoglycan receptor found throughout the placenta syncytiotrophoblast, which is thought to be a key mechanism to produce disease in pregnant women.^61^ Experimental studies with recombinant var2csa, a member of the *P. falciparum* erythrocyte membrane protein 1 (PfEMP1), have shown that it is possible to obtain antibodies inhibiting the adherence of infected erythrocytes to CSA.^62^ Thus, it has been suggested that var2csa would be the ideal candidate for a malaria vaccine especially designed for pregnant women.^63^ However, other receptors could also play an important role in the adherence of the infected erythrocytes to the placenta,^64^ and it is not clear why several studies have failed to show association between different levels of IgGs against CSA binding infected erythrocytes and placental malaria infection.^65^

Establishing a conceptual framework for vaccines to prevent malaria during pregnancy seems a key step. This would require designing of specific target product profiles for mothers, fetus and newborns, which should be accompanied by the crucial allocation of funds to identify specific target antigens and conduct of clinical trials for this particular risk group.^61^

Table 2 summarizes a list of vaccine candidates currently under clinical development.

## Vaccines that Interrupt Malaria Transmission (VIMT)

Embedded within the malERA initiative, a rigorous scientific consultative process to identify knowledge gaps and new tools that will be needed to eradicate malaria globally,^6^ the Consultative Group on Vaccines introduced the concept of vaccines that interrupt malaria transmission (VIMT). This concept would include vaccines whose implementation would entail a significant reduction in the transmission of the disease. For example, vaccines targeting the sexual stages of the parasite in the blood and in the mosquito, those disrupting the development of the parasite inside the vector and those pre-erythocytic and blood stage vaccines that would ultimately reduce sexual and asexual stage parasite rates.^5^ A target product profile (TPP), which is a series of characteristics that a candidate product under development should take into account, has been proposed by this group of experts and is partially shown in table 3. We include here the desired target of a vaccine that aims to have an impact on transmission, although the original TPP proposed also includes a list of minimally acceptable targets for a VIMT.^5^

Among the different research and development priorities concerning VIMT, the need for greater efforts in vaccine development against other species of Plasmodia different to *P. Falciparum* is stressed (mainly *P. vivax*), due to the considerable burden of disease they are responsible for, and because targeting these specific species is considered critical to achieve malaria eradication. Other priorities would be: 1) in the field of vaccine evaluation, harmonizing the available tools for measurement of transmission rates and developing new surrogates for malaria transmission in a community level from individual level immune responses. 2) Further research is needed in the field of transmission dynamics and the biology of malaria parasites, since very little is known on what strains would prevail as transmission falls, or whether there would be a niche for re-emergent parasites. 3) Delivery systems for multicomponent vaccines need to be developed since no studies about compatibilities of these components have been conducted 4) Research on the immune activation by adjuvants will be crucial to design new vaccines that could accompany new recombinant protein-based vaccines. 5) Designing an effective vaccine that could induce humoral immunity against the midgut epithelium of the vector would be of immense utility, since it would prevent human infection by any species of Plasmodium.

## Other Challenges for Malaria Vaccines

Apart from the biological challenges in malaria vaccine development, there are other financial, technical, regulatory and logistical issues which will eventually need to be considered. The lengthy process and high costs of developing a malaria vaccine are clear drawbacks for pharmaceutical companies aiming to develop them. Indeed, it has been estimated that from conception to licensure, a malaria vaccine could need at least 15 years of clinical development and cost around 500 to 1000 million US dollars.^67^ In fact, the RTS,S phase three clinical trial could not have been possible without the partnership of the pharmaceutical industry with non-profit institutions, such us PATH-MVI and the Bill and Melinda Gates Foundation. As vaccine development requires such significant investment, new malaria vaccine candidates would need to show considerable advantages compared to RTS,S before entering clinical development. Consequently, this would probably meanthat any vaccine aimed to be approved by any regulatory agency will probably need to face non inferiority trials, which would further increase the costs. In the ladder of vaccine research, human challenge models are more and more believed to be crucial in order to decide which projects will enter advanced phases of clinical research. In the current context of the global economic crisis, fundraising for malaria vaccine development will not be an easy task. Although malaria is considered one of the “big killers”, this disease will have to compete with others, such as AIDS or tuberculosis, and with other vaccines that have shown great efficacy but have not yet been implemented in many countries in the world.

Implementing a successful childhood vaccination campaign in developing countries is not a trivial undertaking. Engaging all stakeholders of the public health sector is one crucial element, as well as optimizing and strengthening existing delivery methods. Any malaria vaccine targeting those who most need it (children) would need to be staggered within the EPI calendar in order to be technically feasible. Should malaria vaccines need one or more booster doses (a possibility currently being assessed for RTS,S), further delivery approaches will need to be developed. It would be desirable to administer any booster dose together with other EPI vaccines (such us measles at 9 months); however, depending on the immunological profile of the vaccine, specific mass vaccination campaigns would need to be implemented, which will surely have to deal with complex logistical issues in areas where access to health systems is limited, as well as difficulties related to suboptimal cold chains, stock management limitations or undertrained health care personnel, among others.

## Conclusions

Many questions remain to be answered regarding malaria vaccine research. Should the preliminary results of the phase III trial of RTS,S malaria vaccine candidate are confirmed, the goal of licensing the first malaria vaccine will soon become a reality. However, since this vaccine will not be fully efficacious, second generation vaccines with greater protection and perhaps targeting different stages of the parasite cycle (combination vaccines) will be needed. An intervention like a malaria vaccine must surmount formidable biological and immunological challenges during development. Furthermore, logistical, technical and economic realities pose challenges which scientists, health care workers, politicians and communities need to broach successfully for effective implementation, reaching all those who need vaccination, to be realized. Only then, the fascinating prospects of malaria eradication will become more realistic.

## Figures and Tables

**Table 1:** Desirable characteristics of an ideal malaria vaccine

• Be effective preventing clinical disease, severe malaria and transmission in the community
• Be completely safe for young infants and risk populations (pregnant women, people with immune deficiencies or other co-morbidities), with a similar safety profile as other EPI vaccines.
• Provide protection against the 5 species of malaria plasmodia.
• Provide long-lasting immunity
• Be administrable in the first months of life
• Single oral - dose regime compatible with vaccines of the expanded programme on immunization (EPI).
• Easily manufactured, deployed, stored and handled.
• Affordable for governments of low income countries.
• Stable at room temperature.
• Available for travelers of non endemic areas.

**Table 2:** summarizes a list of vaccine candidates currently under clinical development.

Vaccine Project name [Trial registration number]*	Antigen	Adjuvant	Clinical Phase	Trial Sponsor	Comments
***Pre-erythrocytic Projects***					
**RTS,S/AS01E** [NCT00866619]	CSP (207–395) & HepBsAg	AS01	III	GSK	First results phase III already published
**AdCh63/MVA ME-TRAP** [NCT01142765, NCT00890760]	TRAP + ME epitopes (CS, LSA1, LSA3, STARP, EXP1, pb9)		I/IIa, I/IIa	OX	Administered in a Prime-Boost regime
**Adenovirus (Ad35) vectored CS** [NCT00371189 and NCT01018459]	CSP		Ia, Ib	NIAID	
**Polyepitope DNA EP1300** [NCT01169077]	CSP, SSP2/TRAP, LSA-1, EXP-1		Ia	NIAID	DNA vaccine
**Adenovirus (Ad35) and adenovirus 26 (Ad26) vectored CS in heterologous prime-boost regimen** [NCT01397227]	CSP		I/IIa	Crucell	Administered in a Prime-Boost regime.
**Adenovirus (Ad35) vectored CS and RTS,S-AS01 in heterologous prime-boost regimen** [NCT01366534]	CSP		I/IIa	GSK	Administered in a Prime-Boost regime.
**Adenovirus (Ad26) vectored CS; Adenovirus (Ad35) vectored CS**	CSP		I	NIAID	Administered in a Prime-Boost regime.
**PfCelTOS FMP012**	CelTOS (cell-traversal protein for ookinetes and sporozoites)			USAMRMC	
**Pf GAP p52-/p32- (Genetically Attenuated Sporozoites)** [NCT01024686]	Genetically attenuated whole Plasmodium falciparum organism		I/IIa	Seattle Biomedical Research Institute (Seattle Biomed) (USA)	Genetically attenuated whole organism
**PfSPZ: metabolically active, non-replicating malaria sporozoite vaccine** [NCT01001650, NCT01441167]	Sanaria, Inc. (USA)		I/IIa, I		
**PfSPZ Vaccine Radiation Attenuated Sporozoite Vaccine (Single Strains)**	Sanaria, Inc. (USA)		I/IIa		Intravenous administration, whole organism
***Asexual Blood-stage Projects***					
**AdCh63/MVA MSP1** [NCT01003314, NCT01142765]	MSP1		I/IIa, I/IIa	OX	Administered in a Prime-Boost regime.
**EBA175 RII [**NCT00347555, NCT01026246]	EBA175 RII, non-glycosylated	aluminium-phospahte	Ia, Ib	NIAID	
**FMP2.1/AS02A (AMA-1 3D7 E. coli-expressed in AS02A adjuvant)** [NCT00385047, NCT00349713, NCT00358332, NCT00460525]	AMA-1 ( 83(Gly) to 531(Glu))	AS02A	Ia, I/IIa, Ib, Ib, Iib	USAMRMC	
**FMP2.1/AS01B (AMA-1 3D7 E. coli expressed in ASO1B adjuvant)** [NCT00385047]	AMA-1 (the ectodomain amino acids 83(Gly) to 531(Glu))	AS01B	I/Iia	USAMRMC	
**FMP010/AS01B (MSP-1 42 FVO E. coli-expressed in AS01B adjuvant)** [NCT00666380]	MSP-1, p42 subunit	AS01B	Ia	USAMRMC	
**GMZ2**[NCT00397449]	GLURP, MSP3	Alum, DDA- TDB	Ia	EVI	
**GMZ2 field** [NCT00424944, PACTR2010060002033537]	GLURP, MSP3	Alum	Ib, II	AMANET	
**MSP3 (181–276)**	MSP3	Alum, Montanide ISA 720		EVI	
**MSP3 [181–276] field [**NCT00452088, NCT00469651, NCT00652275]	MSP3	Alum	Ib, Ib, IIb	EVI, AMANET	
**SE36** [ISRCTN71619711]	N-terminal of SERA5.	Aluminum hidroxide gel	Ib	BIKEN	
**JAIVAC (MSP1 19/EBA175)** [CTRI/2010/091/0003101]	MSP1 19/EBA175	Mon ISA 720	I	ICGEB/EVI	Mixture of two recombinant proteins
**AMA1-C1/Alhydrogel**^®^**+ CPG 7909** [NCT00984763, NCT00344539, NCT00414336]	AMA1-C1: mixture of two AMA1 proteins, AMA1-FVO and AMA1-3D7, mixed in a 1:1 ratio	Alhydroge l® CPG7909	I/IIa, Ia, Ib	NIAID	
**BSAM-2/Alhydrogel®+CPG 7909** [NCT00889616]	BSAM-2 is a mixture of two MSP1 42 proteins, MSP1 42-FVO and MSP142-3D7; and two AMA1 proteins, AMA1-FVO and AMA1-3D7, mixed in a 1:1:1:1 ratio	Alhydroge l® CPG7910	Ia, Ib	NIAID	
**AdCh63 AMA1/MVA AMA1** [NCT01095055, NCT01142765]	AMA1 (biallelic codon-optimised construct with 3D7, FV0 and common epitopes, with tPA leader sequence)		I, IIa	OX	Administered in a Prime-Boost regime.
**NMRC-M3V-Ad-PfCA NMRC-M3V-Ad-PfCA** [NCT00392015]	CSP (3D7 strain) and AMA1 (3D7 strain)		I/IIa	USAMRMC	Combination vaccine (pre-erythrocytic and blood stage)
**NMRC-M3V-D/Ad-PfCA** [NCT00870987]	CSP (3D7 strain) and AMA1 (3D7 strain)		I/Iia	USAMRMC	Combination vaccine (pre-erythrocytic and blood stage). Prime Boost regime
**CSP, AMA1 virosomes (PEV 301,302)** [NCT00400101, NCT00513669]	AMA-1 and CS mimotopes		Ia, Ib	STI	Combination vaccine (pre-erythrocytic and blood stage). Virosomal vaccine
**ChAd63/AMA MVA/AMA1 +alhydrogel/CPG7909 [**NCT01351948]	AMA1	CPG 7909, alhydrogel	I	OX	Administered in a Prime-Boost regime.
***Sexual stage projects***					
**Pfs25-EPA/Alhydrogel** [NCT01434381]	Pfs25-EPA	Alhydroge l	I	NIAID	Recombinate Protein conjugated to Pseudomonas aeruginosa EPA
***P. vivax Project***					
**VMP001/AS01B** [NCT01157897]	Plasmodium vivax CSP	AS01B	I/IIa	USAMRMC	Chimeric Reombinant Protein

*Alphabetical list of abbreviations*: **AMA**, Apical Membrane Antigen; **AMANET**, African Malaria Network Trust; **BIKEN**, Research Foundation for Microbial Diseases of Osaka University; **CSP**, Circumsporozoite protein; **EPA**, ExoProtein A; **EVI**, European Vaccine Initiative; **GSK**, GlaxoSmithKline Biological; **GLURP**, Glutamate-rich protein; **ME-TRAP**, Multi Epitope - thrombospondin-related adhesive protein; **MVA**, Modified Vaccine Ankara; **MSP**, Merozoite Surface Protein; **NIAID** National Institute of Allergy and Infectious Diseases (USA);**NIH**, National Insitute of Health; **OX**, University of Oxford (UK); **SERA**, Serine Repeated Antigen; **STI**, Swiss Tropical Institute; **USAMRMC**, U.S. Army Medical Research and Materiel Command.

* If starting by NCT, registered at clinicaltrials.gov; by CTRI, at Clinical Trials Registry, India; by ISRCTN, at International Standard Randomized Controlled Trial Number Register; by PACTR, at Pan African Clinical Trials Registry.

**Table 3:** Target Product Profile for Vaccines that Interrupt Malaria Transmission: (Desired target)

• **Indication.** The vaccine should provide protection against *P. falciparum* and *P. vivax* malaria so that R_effective_<1.
• **Target population.** It should be administered to all age groups and populations, including pregnant women, persons with immunodeficiencies, malnourished individuals or other high risk populations
• **Route of administration.** The vaccine would be administered orally or by intramuscular or subcutaneous injection.
• **Product presentation.** It would be available in a single dose auto-disposable compact prefilled device
• **Dosage schedule.** A single dose vaccine that can be administered by either mass administration of clinic based programs.
• **Safety.** The vaccine should have a safety and reactogenicity profile comparable to hepatitis B vaccine.
• **Expected efficacy.** It would reduce R_effective_ below 1.0 in a malaria endemic population and provide protection against *P. falciparum* and *P. vivax* for at least 2 years.
• **Coadministration.** The vaccine should be coadministered with any licensed vaccine without a clinically significant interaction to safety or immunogenicity
• **Shelf life.** The product must have a minimum shelf life of 36 months.
• **Storage.** The product should be stable at room temperature and withstand freeze thawing
• **Product registration.** Product must be WHO prequalified and registered with EMEA and FDA

* Adaptation from the Target Product Profile (TPP) proposed by the malERA Consultative Group on Vaccines.^5^
